# Wastewater-based epidemiology for monitoring enteric viruses: a case study in Valladolid, Spain (2020–2021)

**DOI:** 10.3389/fmicb.2025.1586478

**Published:** 2025-05-30

**Authors:** Lorena Casado-Martín, Marta Hernández, Nadine Yeramian, Maria Jose González-Peña, José M. Eiros, David Rodríguez-Lázaro

**Affiliations:** ^1^Faculty of Science, Area of Microbiology, University of Burgos, Burgos, Spain; ^2^Centre for Emerging Pathogens and Global Health, University of Burgos, Burgos, Spain; ^3^Faculty of Medicine, Area of Microbiology, University of Valladolid, Valladolid, Spain; ^4^AquaVall, ETAP Las Eras, Valladolid, Spain

**Keywords:** enteric viruses, wastewater-based epidemiology (WBE), seasonality, SARS-CoV-2, food safety, one health, pandemic period

## Abstract

Wastewater-based epidemiology (WBE) has been employed for decades and gained renewed significance with the emergence of SARS-CoV-2 at the end of 2019. The incidence of foodborne outbreaks has increased in recent decades, particularly those causing gastroenteritis and diarrhea, which are often of viral origin. However, because many enteric viruses are difficult or uncommon to diagnose, their frequency is often underestimated in comparison to bacterial diseases. WBE provides a valuable alternative for monitoring the presence and evolution of different enteric viruses within a population. This study monitored the major enteric viruses that are potential hazards to public health, including human noroviruses genogroup I and II (NoV GI and GII), human astroviruses (HastV), rotaviruses (RV), and hepatitis A (HAV) and E (HEV) viruses. Viral concentration was performed using an aluminum-based precipitation method, followed by RNA extraction and RT-qPCR quantification. Surveillance was conducted during the COVID-19 pandemics, from October 2020 to October 2021 in Valladolid, Spain, and its surrounding areas. The results showed that both genogroups of noroviruses exhibited the highest normalized concentration levels (5.42 ± 0.08 and 5.44 ± 0.09 Log gc/L, respectively). They were followed by RV (4.41 ± 0.07 Log gc/L) and HastV (6.00 ± 0.11 Log gc/L). Positivity rates were also greater for noroviruses, especially NoVGII (62% and 83.30%, respectively). However, in this case, RV presented a slightly higher positivity rate (46.70%) than HastV (41.30%). Meanwhile, HEV was detected only once (0.67% positivity), and HAV was absent throughout the study period. Additionally, lower concentration levels of the monitored pathogens were detected, compared to later periods, likely because of public health measures implemented during the COVID-19 pandemic. In conclusion, these findings highlight the potential of WBE for the early detection and monitoring of enteric virus outbreaks, particularly during public health crises.

## 1 Introduction

Wastewater-based epidemiology (WBE) has been used for decades to monitor drug and medicine consumption (Zuccato et al., 2000; Ternes, 1998) as well as pathogens like poliovirus (Paul et al., 1939). This approach gained renewed importance with the emergence of SARS-CoV-2 (Ahmed et al., 2020), proving a valuable tool for tracking pathogens and understanding their evolution and transmission patterns within populations. WBE provides critical epidemiological insights, allowing for the monitoring of infectious agents both geographically and temporally (Islam et al., 2024).

*Campylobacter, Salmonella, Yersinia enterocolitica*, Shiga toxin-producing *E. coli*, and *Listeria monocytogenes* have been reported usually as the main zoonotic agents responsible for gastrointestinal illness in Europe. Nonetheless, a large number of diarrhea and gastroenteritis are caused by viruses. The 2022 European annual report on zoonoses states that foodborne outbreaks have increased by 43.9% compared to 2021, with the highest death toll in the past decade. Notably, norovirus was linked to a sizable percentage of these outbreaks, highlighting the significance of viruses as threats to public health (EFSA and ECDC, 2023). According to the Centers for Disease Control and Prevention (CDC), between 1970 and 2020, foodborne outbreaks in the United States caused 2 million illnesses and 2,205 deaths, with viruses responsible for 49% of the cases and 45% of fatalities. These trends can likely to be extrapolated to other developed regions like Europe. The percentage of viral-related total outbreaks, illnesses, hospitalizations, and deaths has risen steadily over the previous 20 years, reaching 55, 68, 35, and 54%, respectively, in the last decade (2011–2020) (Olaimat et al., 2024).

Many of these viruses are difficult to cultivate *in vitro*, and reliable rapid diagnostic tests are often unavailable. Consequently, their impact is likely underestimated compared to bacterial pathogens (O'Shea et al., 2019). Their low infectious dose (as few as 100 viral particles can cause illness) and their ability to trigger rapid outbreaks make them highly effective as etiological agents (Olaimat et al., 2024). Moreover, they are typically transmitted via the fecal-oral route, entering the human body through the gastrointestinal tract and being excreted in large quantities through feces or vomit. Once in the environment, these viruses can remain infectious for months (Boone and Gerba, 2007), making them ideal candidates for monitoring through wastewater analysis.

In the past decade, in regions such as the U.S., Canada, the European Union, and parts of developed Asia, the majority of outbreaks have been attributed to norovirus (Thomas et al., 2013; EFSA and ECDC, 2023; Hashemi et al., 2023), followed by sapovirus, rotavirus, viral hepatitis, adenovirus, and astrovirus (Bosch et al., 2018; Olaimat et al., 2024).

Norovirus is a genus within the *Calciviridae* family, which also includes Sapovirus. These two pathogens are responsible for acute gastroenteritis. Currently, ten different norovirus genogroups have been identified; however only genogroups I and II infect humans (Nov GI and NoV GII), with NoV GII being the most prevalent in the population (Christiane and Kim, 2021). These pathogens are associated with infections across all age groups, although breastfed infants, children under 5 years old, and the elderly are considered at higher risk for severe illness. Currently, no vaccine is available for these infections; however several research projects are working on vaccine development in response to the significant economic impact of these diseases (Bartsch et al., 2016). Rotavirus, belonging to the *Reoviridae* family, is the leading cause of severe gastroenteritis in children under 5 years old, although it can infect individuals of all ages (Omatola and Olaniran, 2022). It is estimated that 95% of children contract the virus within their first 3 to 5 years of life. Currently, two globally implemented vaccines have significantly reduced mortality associated with this pathogen. However, RV remains responsible for more than 200,000 annual deaths worldwide (Organización Panamericana, de la Salud, 2007). Human astrovirus, part of the *Astroviridae* family, is often found in co-infections with other gastrointestinal pathogens such as norovirus and rotavirus (De Benedictis et al., 2011; Jacobsen et al., 2018). This virus primarily infects children under 5 years old, although the elderly and pregnant individuals are also vulnerable. This virus is the second or third leading cause of diarrhea in children, following norovirus and rotavirus (Méndez et al., 2012). To date, no vaccines have been developed against human astroviruses. Finally, HAV and HEV are both responsible for viral hepatitis, although they belong to different families. In the case of hepatitis A, infections are often asymptomatic; however, jaundice occurs in approximately 70% of adult cases (Lemon et al., 2018). HEV, on the other hand, is the main cause of acute hepatitis worldwide (Sridhar et al., 2015), with increased mortality observed in pregnant women and individuals with preexisting chronic liver disease (Kumar et al., 2004; Pérez-Gracia et al., 2017). A vaccine against HEV has been developed but it is only approved in China (O'Shea et al., 2019). Additionally, an antiviral treatment is available, though it is teratogenic (Krzowska-Firych et al., 2018).

Reliable detection of viruses in food matrices remains a challenge for several reasons. First, the isolation and detection methods are often laborious and suboptimal. Second, the low concentration and heterogeneous distribution of the pathogen in food complicates the process. Finally, it is difficult to determine whether the detected viral load correlates with infectious capacity (Barrabeig et al., 2010; Bosch et al., 2018). For these reasons, studying the presence of these pathogens in alternative matrices, such as wastewater, may offer better insights into their behavior. This approach is particularly promising for enteric viruses, which are excreted in large quantities in feces.

The true prevalence of these viral pathogens is often underestimated for several reasons. In general, the frequent asymptomatic course, the similarity of symptoms among them, and the tendency to administer general gastrointestinal treatments in the absence of specific laboratory tests are the main factors contributing to this issue. Additionally, in the case of HEV infections, viral particles are no longer detectable in the blood three weeks after infection, although they can still be observed in feces for up to two additional weeks (Kamar et al., 2014). This, combined with the high number of asymptomatic cases (Olaimat et al., 2024), leads to an underestimation of the HEV significance. Similarly, underreporting of HastV is due to its frequent asymptomatic presentation and, despite its clinical and agricultural relevance, its status as one of the least-studied viruses (Cortez et al., 2017). For all these reasons, along with their significant presence in feces—and consequently in wastewater—the investigation of the prevalence of these viruses in the population through wastewater analysis is particularly valuable. To this end, this study focuses on the surveillance of major enteric viruses that suppose a public health threat and tracks their evolution between October 2020 and October 2021 for the first time in Valladolid (Castilla y León, Spain) and its surrounding areas. A better understanding of the local and sub-local temporal distribution of these viruses during a critical period, such as the first year of the COVID-19 pandemic, was essential for informing institutions not only about the widely monitored SARS-CoV-2, but also about other relevant and often underestimated pathogens. This need became even more critical in the context of an overwhelmed healthcare system, which was primarily focused on saving lives. Additionally, this study reinforces the significant role of WBE as an effective tool for monitoring a wide range of pathogens and supporting public health authorities in making informed decisions.

## 2 Materials and methods

### 2.1 Sampling

**Figure 1:**
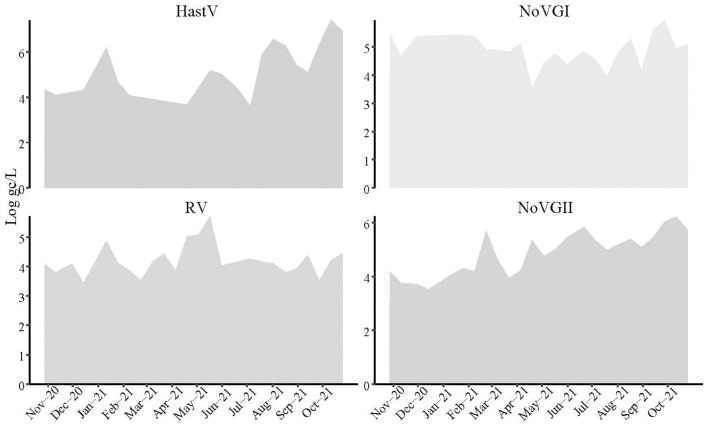
WWTP samples' origin. Spain Map with Castilla y León region colored by light green and Valladolid in dark green (MapChart), and a zoom of the different sampling sites in different colors (done by Google Earth Pro).

From October 2020 to October 2021 wastewater influent samples (*n* = 25) were collected every two weeks at a wastewater treatment plant (WWTP) located in Valladolid, Castilla y León, Spain, that provides services to about 350 000 people. Specifically, six WWTP influents were analyzed at each sampling time from six different locations (Supplementary Table 3) within the province of Valladolid (Figure 1), for a total of 150 samples examined.

During early morning monitoring (7–10 am), one liter of grab wastewater was collected for each sample using sterile, thiosulphate-free PET containers (VWR^®^, Avantor, US). Collected samples were immediately transferred on ice to the laboratory, stored at 4°C, and processed within the first 12 h after collection.

### 2.2 Viral concentration method and nucleic acid extraction

Two hundred milliliters of wastewater were transferred to a sterilized 250 mL PPCO centrifuge bottle (Thermo Fisher Scientific^TM^ Nalgene^TM^ Products, US). Then samples were then inoculated with 2.11 × 10^2^ Infective Units (IU) of Mengovirus (vMC0, CECT, Spain) as process control. All enteric viruses were concentrated using an aluminum-based adsorption-precipitation method (Randazzo et al., 2020). To continue with the protocol, pH was adjusted to 6.0 before precipitation step by adding 1 part 0.9 N AlCl_3_ (Acros organics, Geel, Belgium) solution to 100 parts of sample. Then, pH was readjusted to 6.0, and the sample was mixed using an orbital shaker at 150 rpm for 15 min at room temperature.

The viruses were then concentrated by centrifugation at 1,900 × g for 30 min. The resulting pellet was resuspended in 10 mL of 3 % beef extract pH 7.4 (Lab-Lemco Powder, Oxoid, Thermo Fisher Scientific^TM^, US), transferred to 50 mL PP centrifuge tubes (Corning, US), and shaken for 10 min at 150 rpm. The concentrate was recovered by centrifugation at 1,900 × g for 30 min, and the precipitate was resuspended in 1 mL of PBS (Thermo Fisher ScientificTM, US), following the method described by Randazzo et al. (2020) and Girón-Guzmán et al. (2024b).

Three process controls were included for each collection date analyzed (D'Agostino et al., 2011). A sample process control (SPC), which consisted of 200 mL of autoclaved tap water, inoculated with the same quantity of Mengovirus, and identically to the other samples. This control was used to ensure the accuracy of the concentration, extraction and quantification of nucleic acids (Diez-Valcarce et al., 2011). On the other hand, a negative process control (NSPC) was carried out to detect potential cross-contamination during processing. This control used 200 mL of autoclaved tap water, processed in the same way as the samples, but without the addition of Mengovirus. Moreover, an extraction control was performed, for each collection date, by inoculating 3 mL of the sterilized tap water (the approximate final volume of viral concentrate obtained from each sample) with the same quantity of Mengovirus. This control was only subjected to the extraction and quantification steps, simulating an ideal concentration. It was used to verify the efficiency of the nucleic acid extraction step.

Nucleic acid extraction from wastewater concentrates was performed using QIAmp^®^ Viral RNA Mini Kit (Qiagen, Germany), following the manufacturer's instructions. This step was carried out from 150 μL of concentrate.

### 2.3 Enteric virus quantification by RT-qpcr

Six public health-relevant enteric viruses were studied in each wastewater sample described in section 2.1, specifically NoV GI and NoV GII, human astroviruses (HastV), hepatitis A and E virus (HAV, HEV), and human rotaviruses (RV). To assess for inhibition, each RT-qPCR assay was performed in duplicate wells using both undiluted RNA and a 10-fold dilution. In the same plate, duplicates of the SPC, NSPC, and extraction control were also included as non-template controls. For each virus and assay, a standard curve was generated using commercially available quantitative synthetic RNA (Supplementary Table 1).

Enteric virus levels in the samples were determined by one-step RT-qPCR reactions, using TaqMan^TM^ Fast Virus 1-Step Master Mix (Applied Biosystems, US), in a final volume of 10 μL, with 2.5 μL of either undiluted, 10-fold diluted, or synthetic RNA. All experiments were conducted using a QuantStudio5 thermocycler (Applied Biosystems, US).

Primers and probes sequences are provided in Supplementary Table 2, and their final concentration, and the thermocycler conditions were based on the references cited in the table. Except the reverse transcription step, which was shortened according to the TaqMan^TM^ Fast Virus 1-Step Master Mix instructions. This modification, supported by Gunson et al. (2006), significantly reduced the reaction time without compromising sensitivity.

To estimate viral titers, cycle threshold (Cts) values ≤ 40, obtained using QuantStudio^TM^ Design & Analysis software (v1.5.1), were converted into genomic copies per liter (gc/L) using the corresponding standard curve, dilution factors, and recovery rates. For comparative purposes, the fixed threshold was manually adjusted close to the values suggested by the software. Specifically, the threshold value was set at 0.04 for all enteric viruses, except for HAV, where it was set at 0.08.

Inhibition was assessed by comparing the average viral titers from duplicate wells of undiluted RNA and 10-fold diluted RNA. Inhibition was confirmed if the difference in viral titers was >0.5 Log_10_ and, in such cases, viral levels were inferred from the 10-fold RNA diluted values.

Quantification was carried out by using the standard curve obtained in each experiment, with the aim of considering the intrinsic variability of RT-qPCR and obtaining more reliable results.

### 2.4 Data validation and normalization

All the assays were validated through different steps. Negative controls were first checked to be negative; if not, RT-qPCR was repeated. Second, considering the inherent variability between experiments, the standard curve was calculated and, its quality parameters (efficiency, r-adjustment, etc.) were assessed to confirm they were within acceptable ranges. Once results were validated, the quantification was carried out as described in Section 2.3 and then results were normalized. Normalization is a crucial aspect of WBE, to compare prevalence in different areas with a different population density and periods (Foladori et al., 2024; Polo et al., 2020).

In this study, data were normalized per 100,000 inhabitants, thanks to the population size of each area provided by Wastewater Treatment Plant (WWTP; Supplementary Table 3). Normalizing using the population size supplied by the sewage- referred to as the “*de facto*” population may be enough and recommended in a non-tourist site (CDC, 2023; Daughton, 2012), if no other information, such as hydraulic and/or chemical parameters, is available.

### 2.5 Plots and statistical analysis

All downstream plots were carried out with R (version 4.4.0) using ggplot2 (v. 3.5.1) package.

## 3 Results and discussion

### 3.1 General evolution of enteric virus loads

**Figure 2:**
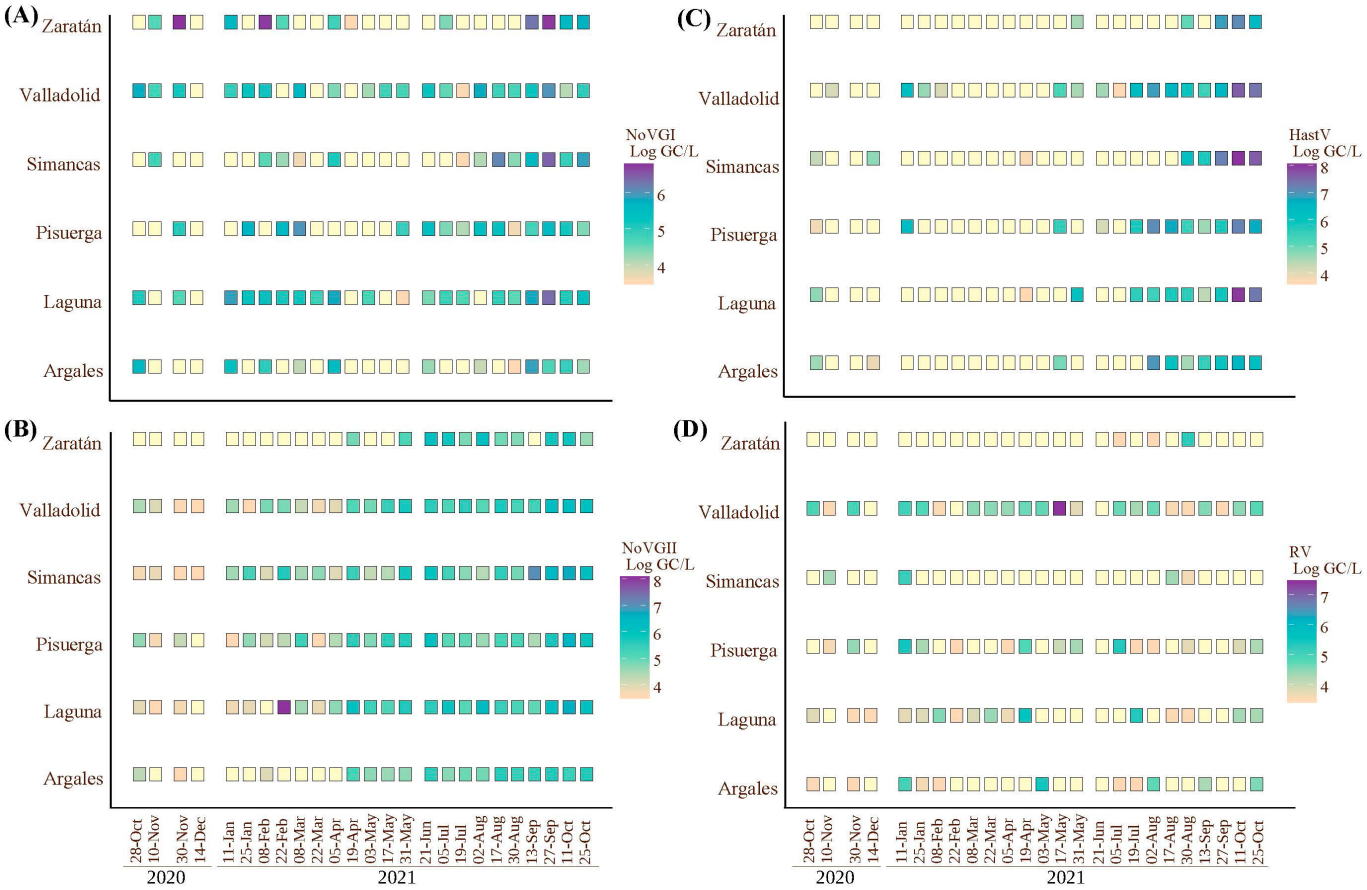
Temporal evolution of human astroviruses, rotaviruses, and noroviruses expressed as the average of the six sampling areas as logarithm of the genome copies per liter (Log gc/L) and 100,000 inhabitants.

Every 2 weeks, six different influent samples from a wastewater treatment plant (WWTP) located in Valladolid, Castilla y León, Spain, were analyzed by RT-qPCR to monitor the presence of six enteric viruses of public health relevance NoV GI, NoV GII, RV, HastV, HAV, and HEV. The viral load expressed as the average of the six samples analyzed on each sampling date and quantified in logarithmic units of genome copies (Log gc/L) per 100,000 inhabitants, fluctuated significantly over time (Figure 2).

Both genotypes of human noroviruses (NoV GI and NoV GII) reached concentrations ranging from 3.02 to 7.95 and 2.98 to 8.65 Log gc/L, respectively; while HastV exhibited slightly higher mean concentration, particularly toward the end of 2021 (~7 Log gc/L). In contrast, human rotavirus (RV) displayed lower levels compared to the other three viruses (4.41 Log gc/L), with a notable increase observed in May 2021 (Table 1).

**Table 1:** Descriptive Statistics from the six areas studied and all sampling times.

	**NoV GI**	**NoV GII**	**HastV**	**RV**	**HEV**
Mean	5.42	5.44	6.00	4.41	4.35
St. error	0.08	0.09	0.11	0.07	0.06
Min.	3.02	2.98	3.05	2.98	4.30
Max.	7.95	8.67	9.28	6.88	4.40
Positivity rate	62.00%	83.30%	41.30%	46.70%	0.67%

Results are expressed as Log gc/L per 100,000 inhabitants.

Noroviruses were consistently detected throughout the year, except for NoV GI, which was absent on December 14, 2020. Furthermore, since May 2021, both genotypes have shown a similar evolutionary trend, albeit at different concentration levels, with NoV GII generally presenting higher concentrations than NoV GI. An increase in NoV GII concentrations was observed in February 2021, followed by four additional peaks in April, June, August, and October, with the second and fourth peaks exhibiting the highest concentrations. Similarly, NoV GI exhibited increases at the beginning of April, preceding the peak of NoV GII, while the other peaks occurred either simultaneously or up to 2weeks before NoV GII. Those facts suggest a co-evolutionary pattern between the two genotypes.

On the other hand, RV exhibited a continuous presence throughout the year, apart from June 2021, when it was absent. Elevated concentration levels were recorded between April and May 2021, with a peak occurring on May 17. Additionally, another increase was detected at the beginning of 2021. Conversely, HastV showed a more intermittent presence, with concentration levels frequently falling below the detection limit. Gaps were observed at the end of November 2020 and between February and May, with a slight appearance on April, 2021. Three significant increases were detected in January, August (slightly earlier than the norovirus peak), and again, on October 2021, with the latter showing the most substantial increase.

Finally, all samples analyzed were negative for HA, whereas for HEV, samples were either negative or exhibited viral loads below the detection limit, except for one sample from Laguna de Duero on August 2021, which showed a viral load of 4.35 Log gc/L.

### 3.2 Seasonality of enteric viruses in each area

**Figure 3:**
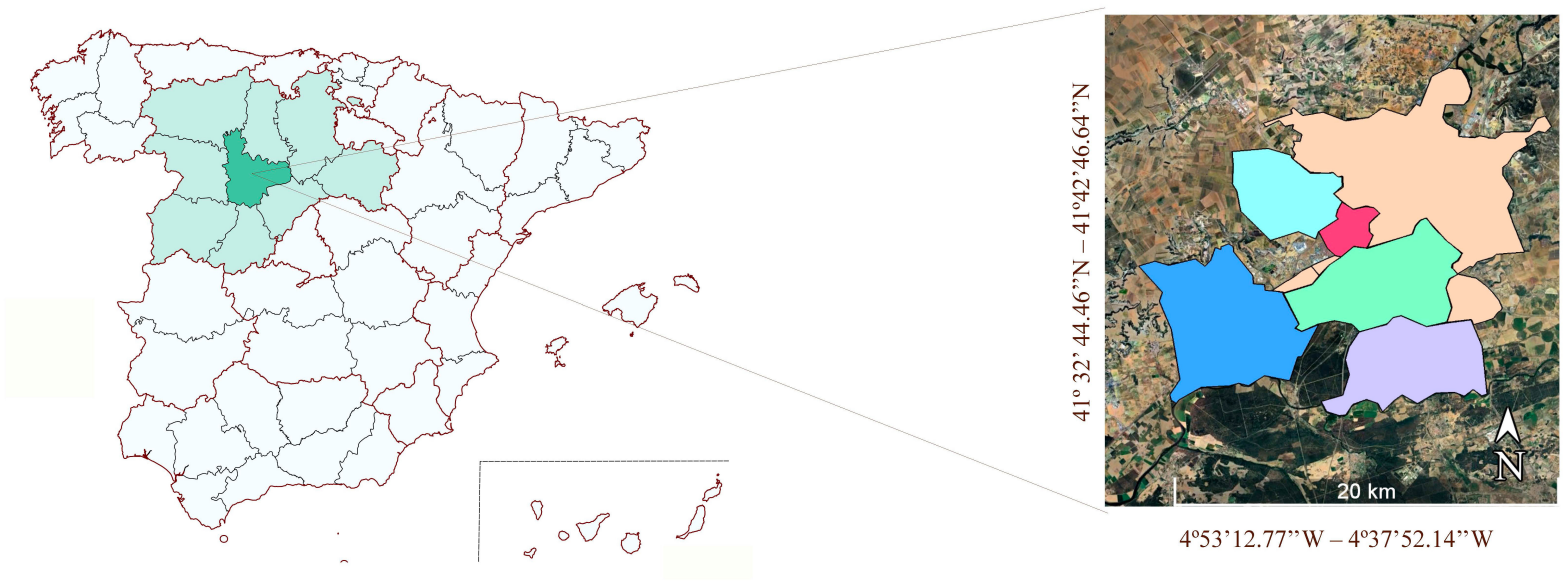
Viral load of NoV GI (A), NoV GII (B), HastV (C) and RV (D) in logarithmic genome copies per liter and 100,000 inhabitants, for each area and sampling date (same date for all viruses).

Higher levels of NoV GI and NoV GII were detected in all studied areas toward the end of the year studied, particularly from June 2021 onwards (Figure 3). In general, NoV GI exhibited more heterogeneous prevalence across the year in all areas compared to NoV GII which remains more stable. However, in Zaratan and Argales, the prevalence of NoV GII was irregular until June and April, respectively. More specifically, NoV GI showed isolated peaks in Zaratan area during November 2020, February 2021, and from September to October 2021. Notably, the highest concentration was detected on September 2021, in Zaratan, aligning with similar trends in Simancas and Laguna. Viral loads were either below the detection limit or ranged between 4.00 and 6.00 Log gc/L for most of the rest of the samples.

Regarding NoV GI, Zaratan and Argales had 52.00% of the samples showing viral loads below the detection limit, followed by Simancas with 48.00%. In the case of NoV GII, a notable increase was detected on February 2021 (Figure 3). Additionally, the increase observed in September was also evident, particularly in Simancas samples.

In case of NoV GII, Zaratan and Argales showed several samples with viral loads below the detection limit, with 56.00 and 32.00% of the samples, respectively. However, the proportion of negative samples for this genogroup was significantly smaller compared to NoV GI, as areas like Simancas and Valladolid did not exhibit this phenomenon. The maximum viral load for NoV GII reached approximately 8.00 Log gc/L, exceeding that of NoV GI by more than one logarithmic unit.

Human astrovirus exhibited homogeneous behavior across all sampling areas. However, it displayed a heterogeneous pattern over time. As shown in Figure 3, high levels of astrovirus viral loads were detected in August 2021, and October 2021, with concentrations ranging between 6.00 and 8.00 Log gc/L. Conversely, in the remaining months, levels were either low or below the detection limit. Interestingly, during the same period the previous year, viral loads were generally below the detection limit or around 4.00 and 5.00 Log gc/L. This suggests that human astrovirus demonstrated significant variability, with low overall prevalence but high concentrations when it was present. In fact, between 52.00 and 80.00% of the samples showed concentrations below the detection limit, with the exception of Valladolid, where 64.00% of the samples had levels over the detection threshold.

On the other hand, concentration levels of human rotavirus were lower than those of the other enteric viruses studied, with the highest concentrations reaching around 7.00 Log gc/L, while the other three viruses typically ranged between 8.00 and 9.00 Log gc/L. This indicates a lower prevalence of rotavirus within the population. More specifically, the Valladolid inlet exhibited higher levels of RV throughout the study period, particularly on May 2021, when levels exceeded 7.00 logarithmic units. The distribution of RV levels was more variable across areas and sampling dates, making it more difficult to discern clear patterns compared to the other three viruses. Notably, while several areas, i.e. Zaratan, Simancas, and Argales, exhibited high rates of negative samples (92.00, 84.00, and 56.00%, respectively), some peaks, heterogeneously distributed across areas and dates, could be sawn, like those observed on August 30 in Zaratan, May in Valladolid, June in Simancas, April and July in Laguna, and May in Argales.

## 4 Discussion

Clinical surveillance of most enteric viruses is infrequent because symptomatology is very similar between the responsible pathogens and specific tests are rarely done. In this study, we carried out a wastewater-based epidemiology study with the aim of observing the prevalence and temporal patterns of six public health-relevant enteric viruses present in six different areas in the province of Valladolid, Castilla y León, Spain.

### 4.1 Seasonality of the enteric viruses

To gain a more precise understanding of the seasonality of the enteroviruses studied, it would be necessary to extend the duration of the study, allowing the detection of repetitive patterns over multiple years. Nevertheless, differences in the prevalence of certain viruses across seasons were observed. The two norovirus genogroups exhibited a slight opposing tendency in their behavior, even though their prevalence stayed largely constant throughout the year. NoV GI presented slightly higher average concentrations during autumn and winter, ranging between four and six logarithmic units, consistent with previous findings (Farkas et al., 2018). In contrast, NoV GII reached its highest concentration during spring and summer. Our finding for both genogroups were similar to those detected in Chile during the spring and summer of 2021 (Plaza-Garrido et al., 2023). Overall, the mean concentrations of NoV GI were somewhat higher than those observed in another region of Spain 2 years earlier (Cuevas-Ferrando et al., 2022) and 1 year later (Girón-Guzmán et al., 2024b). Conversely, NoV GII concentrations were comparable to those reported in the years preceding the pandemic (Cuevas-Ferrando et al., 2022), though they were two logarithmic units lower than levels observed by Girón-Guzmán et al. (2024b).

During the study period, HastV showed a slightly higher prevalence in spring and summer. In the autumn and winter of 2020, this virus exhibited a very low presence, contrasting with the same period in 2021. The mean concentration of HastV during the analyzed months was one logarithmic unit lower than that recorded by Girón-Guzmán et al. (2024b).

Rotavirus (RV), on the other hand, did not exhibit a marked seasonality but showed sporadic peaks. This is notable as, in temperate climates such as Spain, this virus is generally more prevalent in winter and spring (CDC, 2023). This behavior could be attributed to the fact that RV primarily affects children under 5 years old, whose waste is often managed via diapers, preventing its entry into the wastewater system. The detected RV levels align with those reported 1 year earlier (Cuevas-Ferrando et al., 2022), while they were lower than those observed 1 year later in other city in the same country (Girón-Guzmán et al., 2024b). In general, RV presence was stable across all areas except for Zaratan and Simancas, likely due to the low population representation in these regions.

Overall, during the year of the study, viral concentrations detected were lower than those observed in other studies 1 year later, particularly for rotavirus, except for NoV GI. This difference may be attributed to the fact that the measures imposed during COVID-19 pandemic impacted the transmission of noroviruses (Keaveney et al., 2022) in various regions, including England (Douglas et al., 2021), Australia (Bruggink et al., 2021), and the United States (Nachamkin et al., 2021). Consequently, when measures were relaxed, 1 year after the beginning of the pandemic, the increase of concentration levels of human noroviruses was observed potentially associated to the increase of the virus transmission along with an immunity debt, generated for the lower contact of population with those pathogens through 1 year.

### 4.2 Comparison between areas

The area referred to as Valladolid was expected to show higher virus concentrations due to its representation of a larger population (Supplementary Table 3). However, the results revealed the opposite. Despite being the area with the lowest percentage of samples below the detection limit, it exhibited the lowest virus concentrations. This may be due to the larger volume of wastewater, both urban and industrial, which could dilute the genetic material of the studied viruses or make the sample composition even more heterogeneous, reducing methodological efficiency.

Zaratan had the lowest average recovery rates (28.47%) but not so far from others like Argales (33.11 %), Pisuerga (30.24%), or Valladolid (32.92%). In addition, Zaratan also showed the highest negativity rates, i.e., the percentage of samples below the detection limit, exceeding 50% in all the four viruses that could be quantified. This could be attributed to relatively small population of Zaratan (6,400 inhabitants), that can reduce the viral seed and consequently, the viral load.

Argales also exhibited high negativity percentages: 52.00% for NoV GI, 32.00% for NoV GII, 60.00% for HastV, and 56.00% for RV. This outcome could be linked to the fact that, although it is the third most populous area, the sample includes both urban and industrial wastewater, introducing a significant dilution factor.

Simancas, the area with the smallest monitored population, also showed high negativity percentages: 48.00% for NoV GI and up to 68.00% and 84.00% for astrovirus and rotavirus, respectively. However, notably, all samples for NoV GII were positive and showed the highest recorded concentrations throughout the study period (an average of 6.22 Log gc/L per 100,000 inhabitants).

Finally, Pisuerga and Laguna showed moderately high positivity levels, ranging between 60.00% and 96.00% for all viruses except for human astrovirus, where the percentages were lower (48.00% and 44.00%, respectively).

### 4.3 Positivity enterovirus rates

NoV GII has the highest positivity rate (83.30%) followed by NoV GI of the same virus type (62.00%), rotavirus, human astrovirus, and HEV. This is consistent with previous research showing that, HastV is the least common enteric virus detected, excluding HEV and HAV (Fu et al., 2023).

HastV, in turn, have the highest average concentrations (6.00 Log gc/L per 100,000 inhabitants) but the largest percentage of negativity (58.70%). This is because, although they are undetectable in many cases, when they are present, they occur at high concentrations, leading to the marked peaks observed in Figure 1. From this result, it can be inferred that the impact of this pathogen on the population is characterized by significant peaks of infection, even when it remains virtually absent or undetectable for the rest of the time using the methodology employed.

The positive rate for the HEV is low. However, this value is lower than what was found during the same period 1 year later in a different location of Spain, although the average levels recorded are slightly similar (Girón-Guzmán et al., 2024b). This low positivity aligns with longer-term studies where HEV also displayed low prevalence (Takuissu et al., 2022). Notably, the only sampling event in which HEV was quantifiable occurred on August 2021, coinciding with overall positivity across all areas for the four predominant enteric viruses.

All samples tested were negative for the HAV, suggesting that there were no outbreaks of this infection during the study period. This result is consistent with prior studies reporting low percentages of positivity, such as the 6.00% observed by Girón-Guzmán et al. (2024a) and the 8.42% reported by Takuissu et al. (2023). Note that many studies included longer time spans, such as the 2015 HAV outbreak. In contrast with another region of the country where the positivity rate was 3.77% during the same period a year later (Girón-Guzmán et al., 2024b), that the lower ratio of positives in this instance is thought to be caused by the consumption of contaminated food such as fruits, raw vegetables, or bivalve mollusks (Spanish Food Safety and Nutrition Agency, 2022). According to the 2020 Food Consumption Report in Spain (Ministry of Agriculture, Fisheries, and Food, 2021), Valladolid is an inland city with a seafood consumption rate of 0.85 kg/person/year, which is notably lower than coastal regions like Valencia (1.66 kg/person/year). For this reason, the low prevalence of HAV in the studied region is reasonable.

Overall, the positive rate for the enteric viruses studied was lower than what was documented at the end of the pandemic, when restrictions were relaxed. This could be attributed to the fact that restrictions implemented to contain the transmission of SARS-CoV-2 also influenced transmission of enteric viruses. However, another explanation could be associated to that the samples analyzed in this study include both urban and industrial wastewater which dilutes the viral load.

Finally, it is important to consider that the quantification of viral concentrations is always influenced by the methodology employed, as well as the heterogeneous nature of wastewater samples. Factors such as precipitation, fecal load, and the presence of organic or inorganic matter can affect any stage of the process, from concentration and extraction to quantification. Additionally, rather than being composite, the samples are collected as grab samples, meaning it represents the specific conditions at the time of collection. For this reason, results should be interpreted cautiously when attempting to draw broader conclusions.

## 5 Conclusions

This study demonstrates the usefulness of wastewater-based epidemiology for monitoring several enteric viruses, providing insights into their prevalence and temporal evolution. The knowledge gained through this approach offers a better understanding of the health status of the population regarding these often clinically underestimated pathogens. This information is of significant relevance and interest for public health, particularly in recent years.

The findings demonstrate that the monitored human enteric viruses ranked in prevalence from highest to lowest as follows: NoV GII, NoV GI, RV, HastV, and HEV. To comprehensively determine the seasonality of these viruses, a longer monitoring period would be required. Nonetheless, the results from this 1-year study indicate a slight seasonal pattern for NoVs and HastVs. RVs exhibited a relatively stable presence throughout the year, punctuated by occasional peaks. In contrast, HastVs displayed periods of undetectable concentrations using the applied methodology but appeared at very high levels when it was present. HEV was the least prevalent, and HAV was completely absent.

Finally, the overall presence of these enteric viruses was lower than that observed during the same period 1 year earlier in other regions. This may be explained by the public health restrictions implemented to prevent the transmission of SARS-CoV-2, which likely impacted on the dissemination of other viruses, including the enteric viruses monitored in this study. This observation aligns with findings from other studies and is further supported by the increase detected in most monitored enteric viruses, except hepatoviruses, during the final weeks of the analysis. This increase coincided with the relaxation of COVID-19 measures and the initiation of the vaccination campaign, during which a significant proportion of the Spanish population received at least one dose of the vaccine during by that time.

## Data Availability

The raw data supporting the conclusions of this article will be made available by the authors, without undue reservation.
