# Supplementary material for: Wastewater-based epidemiology for monitoring enteric viruses: a case study in Valladolid, Spain (2020–2021)
Source: Front Microbiol. 2025 May 30;16:1586478. doi: 10.3389/fmicb.2025.1586478 (PMC12162560; doi:10.3389/fmicb.2025.1586478)
Supplement: Supplementary file 1 [file Table_1.docx]

Supplementary Material

TABLE S1: Exogenous standard material references from ATCC.

| Virus | Reference |
| --- | --- |
| Norovirus GI | Quantitative Synthetic Norovirus G1 (I) RNA (ATCC VR-3234SD) |
| Norovirus GII | Quantitative Synthetic RNA from Norovirus G2 (II) (ATCC VR-3235SD) |
| Human astrovirus | Quantitative Synthetic RNA from Astrovirus (ATCC VR-3238SD) |
| Hepatitis A | Quantitative Synthetic DNA from Hepatitis A virus (ATCC VR-3257SD) |
| Hepatitis E | Quantitative Synthetic RNA from Hepatitis E virus (ATCC VR-3258SD) |
| Rotavirus | Quantitative Genomic RNA from Rotavirus A strain Wa (TC adapted) (ATCC VR-2018DQ) |

TABLE S2: Oligos and TaqManTM probes for enteric viruses studied.

| Virus | Primer name | Primer/probe sequence 3’-5’ | Reference |
| --- | --- | --- | --- |
| NoV GI | QNIF4 | CGC TGG ATG CGN TTC CAT | (ISO 15216-1:2017, 2017) |
|  | NV1LCR | CCT TAG ACG CCA TCA TCA TTT AC |  |
|  | NVGG1p | FAM-TGG ACA GGA GAY CGC RAT CT-BHQ1 |  |
| NoV GII | QNIF2 | ATG TTC AGR TGG ATG AGR TTC TCW GA | (ISO 15216-1:2017, 2017) |
|  | COG2R | TCG ACG CCA TCT TCA TTC ACA |  |
|  | QNIFs | FAM-AGC ACG TGG GAG GGC GAT CG-BHQ1 |  |
| HastV | AstVorf1b+ | AAG CAG CTT CGT GAC TCT GG | (Sano et al., 2010) |
|  | AstVorf1b- | AGC CAT CAC ACT TCT TTG GTC |  |
|  | AstVorf1bp | FAM-AGA GCA ACT CCA TCG CAT TT-BHQ1 |  |
| HAV | HAV68 | TCA CCG CCG TTT GCC TAG | (ISO 15216-1:2017, 2017) |
|  | HAV240 | GGA GAG CCC TGG AAG AAA G |  |
|  | HAV150 | FAM-CCT GAA CCT GCA GGA ATT AA-MGB |  |
| HEV | HEVj_FW | GGT GGT TTC TGG GGT GAC | (Jothikumar et al., 2006) |
|  | HEVj_RV | AGG GGT TGG TTG GAT GAA |  |
|  | HEVj_P | FAM-TGA TTC TCA GCC CTT CGC -BHQ1 |  |
| RV | JVKF | CAG TGG TTG ATG CTC AAG ATG GA | (Jothikumar et al., 2009) |
|  | JVKR | TCA TTG TAA TCA TAT TGA ATA CCC A |  |
|  | JVKP | FAM-ACA ACT GCA GCT TCA AAA GAA GWG T- BHQ1 |  |

TABLE S3: Population size per sample (Area). Data provided by WWTP.

| Area | Inhabitants |
| --- | --- |
| Valladolid | 350,000 |
| Zaratan | 6,400 |
| Simancas | 5,500 |
| Argales | 40,000 |
| Laguna | 22,700 |
| Pisuerga | 80,000 |
